# MIR34A modulates lens epithelial cell apoptosis and cataract development via the HK1/caspase 3 signaling pathway

**DOI:** 10.18632/aging.204854

**Published:** 2023-07-06

**Authors:** Lujia Feng, Yantao Wei, Yimeng Sun, Linbin Zhou, Shaowei Bi, Weirong Chen, Wu Xiang

**Affiliations:** 1 State Key Laboratory of Ophthalmology; Zhongshan Ophthalmic Center, Sun Yat-sen University; Guangdong Provincial Key Laboratory of Ophthalmology and Visual Science; Guangdong Provincial Clinical Research Center for Ocular Diseases, Guangzhou 510060, China; 2 Shenzhen Eye Hospital, Jinan University, Shenzhen Eye Institute, Shenzhen 518040, China

**Keywords:** cataract, MIR34A, HK1, caspase 3, apoptosis

## Abstract

Cataracts are the leading cause of blindness in the world. Age is a major risk factor for cataracts, and with increasing aging, the burden of cataracts will grow, but the exact details of cataractogenesis remain unclear. A recent study showed that microRNA-34a (MIR34A) is involved in the development of cataracts, but the underlying pathogenesis remains obscure. Here, our results of microRNA target prediction showed that hexokinase 1 (HK1) is one of the genes targeted by MIR34A. Based on this finding, we focused on the function of MIR34A and HK1 in the progress of cataracts, whereby the human lens epithelial cell line SRA01/04 and mouse lens were treated with MIR34A mimics and HK1 siRNA. We found that HK1 mRNA is a direct target of MIR34A, whereby the high expression of MIR34A in the cataract lens suppresses the expression of HK1. *In vitro*, the upregulation of MIR34A together with the downregulation of HK1 inhibits the proliferation, induces the apoptosis of SRA01/04 cells, and accelerates the opacification of mouse lenses via the HK1/caspase 3 signaling pathway. In summary, our study demonstrates that MIR34A modulates lens epithelial cell (LEC) apoptosis and cataract development through the HK1/caspase 3 signaling pathway.

## INTRODUCTION

Cataracts, which are the opacification of lenses in the eye, affect 95 million people worldwide and are the leading eye disease causing blindness in many countries [1]. Age is a major risk factor for cataracts, and with increasing aging, the burden of cataracts will grow. In addition to the vision loss caused by cataracts, there are many complications associated with cataracts, such as glaucoma [2], uveitis [3], and corneal endothelial decompensation [4]. Fortunately, this visual impairment is treatable, and the surgical results are often entirely satisfactory for most patients. Unfortunately, surgery is not accessible for all patients due to high expense and the risk of related risks and complications (such as posterior capsule opacification, uveitis, glaucoma [5]) especially in developing countries. Lens epithelial cells (LECs) have been identified as an essential biological component in the pathogenesis of noncongenital cataracts in animals and human beings [6]. Previous research has demonstrated that abnormal behaviors in LECs promote cataract development and complications after cataract surgery [7–10]. However, definite biochemical mechanisms underlying cataract formation have not been completely elucidated and, to date, there are no practical methods to prevent the occurrence of cataracts. Therefore, there is an urgent need to further clarify the pathophysiological mechanisms underlying abnormal changes in LECs in understanding this devastating disease and for developing prevention strategies and new treatments.

MicroRNAs are small single-stranded noncoding RNA molecules that are widely expressed in different organisms and organs and involved in the post-transcriptional regulation of gene expression. MicroRNAs are reported to be involved in various pathophysiological processes, such as cell proliferation, apoptosis, and senescence [11]. Naturally, they participate in different diseases, for example, cancer [12], age-related disease [13, 14], and immune-related disease [15]. MicroRNA-34A (MIR34A) is reported to partake in a multitude of physiological and pathological processes, including age-related diseases, such as age-related vasculature alteration [16], degenerative Cardiovascular Conditions [17], Alzheimer's disease [18], age-related hearing loss [19], and so on. In our previous research, we found that MIR34A is most highly expressed in the epithelium of cataractous lenses [20]. However, whether MIR34A participates in cataractogenesis has not been fully addressed.

Hexokinase (HK) plays an essential role in the catalytic process of glucose metabolism as the only rate-limiting enzyme in glycolysis. Therefore, sufficient expression of HK is necessary for cells to carry out routine physiological functions. Abnormal HK expression leads to the development of numerous diseases, with previous studies showing that HK is upregulated in many different types of tumors [21, 22]. It is widely accepted that HK has four isomers, whereby only Hexokinase 1 (HK1) and Hexokinase 2 (HK2) are expressed in the human lens, accounting for 71% and 29%, respectively [23]. It is known that the lens is an avascular organ, and its nutrition originates from aqueous humor. In addition, the lens is in an oxygen-deficient environment [24, 25] and, accordingly, most ATP in the lens is produced by anaerobic glycolysis, with only a small proportion derived from aerobic respiration [26]. This means HK1 and HK2 may play essential roles in the physiological function of the human lens. In this study, we used microRNA target prediction software, which showed the presence of a binding site for MIR34A on HK1 but not on HK2. Therefore, we further investigated whether MIR34A participates in human LEC energy metabolism via HK1.

## RESULTS

### MIR34A is overexpressed in cataract, inhibiting proliferation and inducing apoptosis of SRA01/04 cells

**Figure 1:**
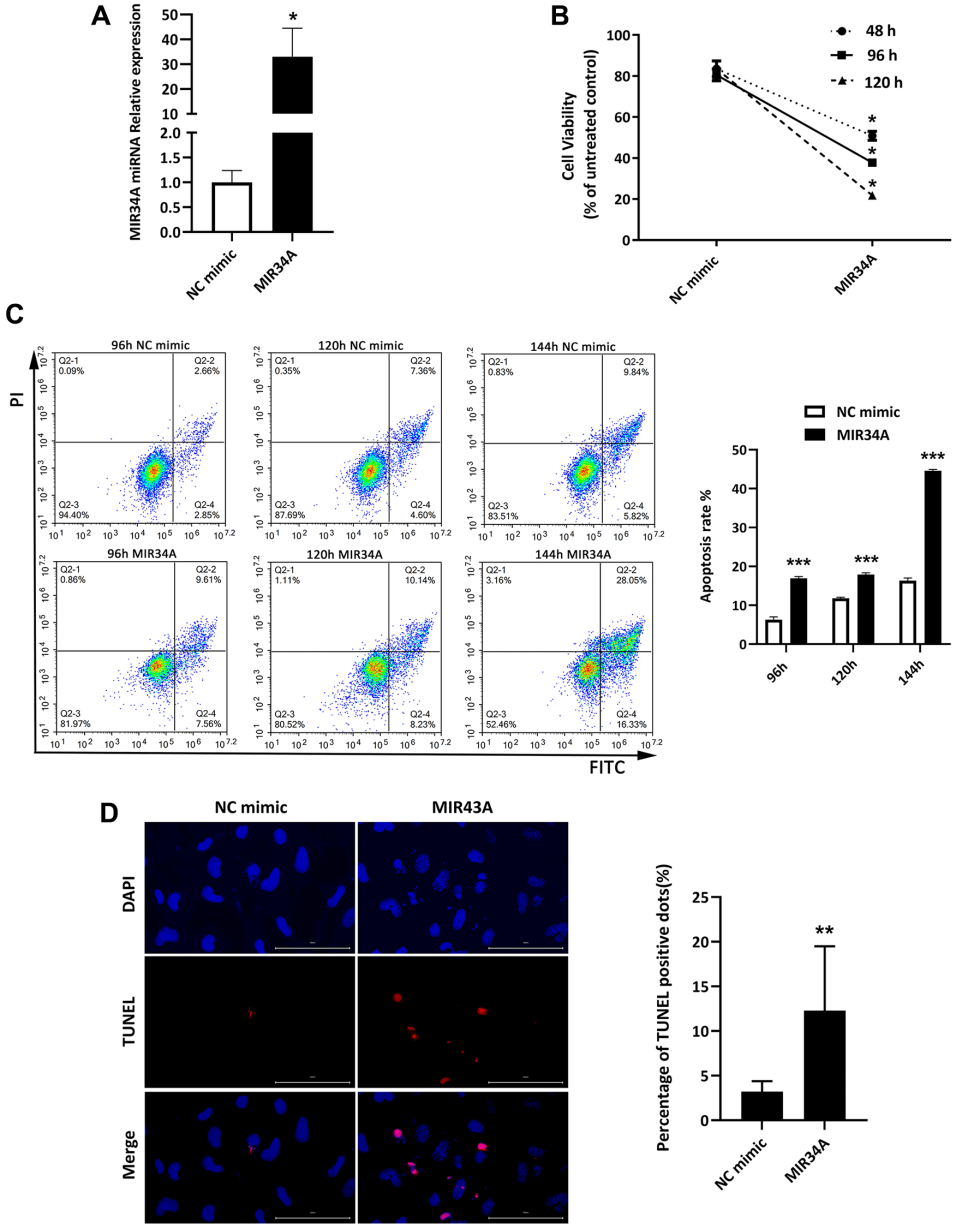
**MIR34A is overexpressed in cataracts, inhibiting proliferation and inducing apoptosis of SRA01/04 cells.** (**A**) RT-qPCR analysis was performed to detect the expression of MIR34A after MIR34A mimics were transfected into SRA01/04 cells. (**B**) SRA01/04 cells were performed as A, the viability of cells was assessed at 48 h, 96 h, and 120 h by CCK-8 after transfected. (**C**) SRA01/04 cells were performed as A, and the cell apoptosis was assessed at 96 h, 120 h, and 144 h by flow cytometry. (**D**) SRA01/04 cells were performed as A, the TUNEL assay was performed to detect the apoptosis at 120 h. Scale bars: 100 μm. ^*^P < 0.05; ^**^P < 0.01; ^***^P < 0.001.

Our previous chip data showed MIR34A is highly expressed in age-rated cataracts compared to transparent lenses [20]. This result indicates MIR34A might be involved in the process of cataract development. To verify this hypothesis, we used MIR34A mimics to upregulate the expression of MIR34A, then reverse transcription- real-time polymerase chain reaction (RT-qPCR) analysis was performed to detect the expression of MIR34A. After treatment with MIR34A mimics, MIR34A expression was significantly increased (Figure 1A). We then used a cell counting kit-8 assay (CCK-8) assay to evaluate the proliferation of SRA01/04 cells. The results show that MIR34A overexpression significantly reduced SRA01/04 cell proliferation at 48, 96, and 120 h (Figure 1B). To analyze the effects of MIR34A on the apoptosis of SRA01/04 cells, two independent techniques of flow cytometry and terminal deoxynucleotidyl transferase dUTP nick end labeling (TUNEL) assay were used. The results of both showed that the upregulation in MIR34A expression significantly increased the apoptotic rate as determined by flow cytometry (Figure 1C) as well as observed according to the number of TUNEL-positive SRA01/04 cells (Figure 1D). These results suggest that miR-34a is overexpressed in cataracts, inhibiting proliferation and inducing apoptosis of SRA01/04 cells.

### MIR34A directly targets and represses the expression of HK1

**Figure 2:**
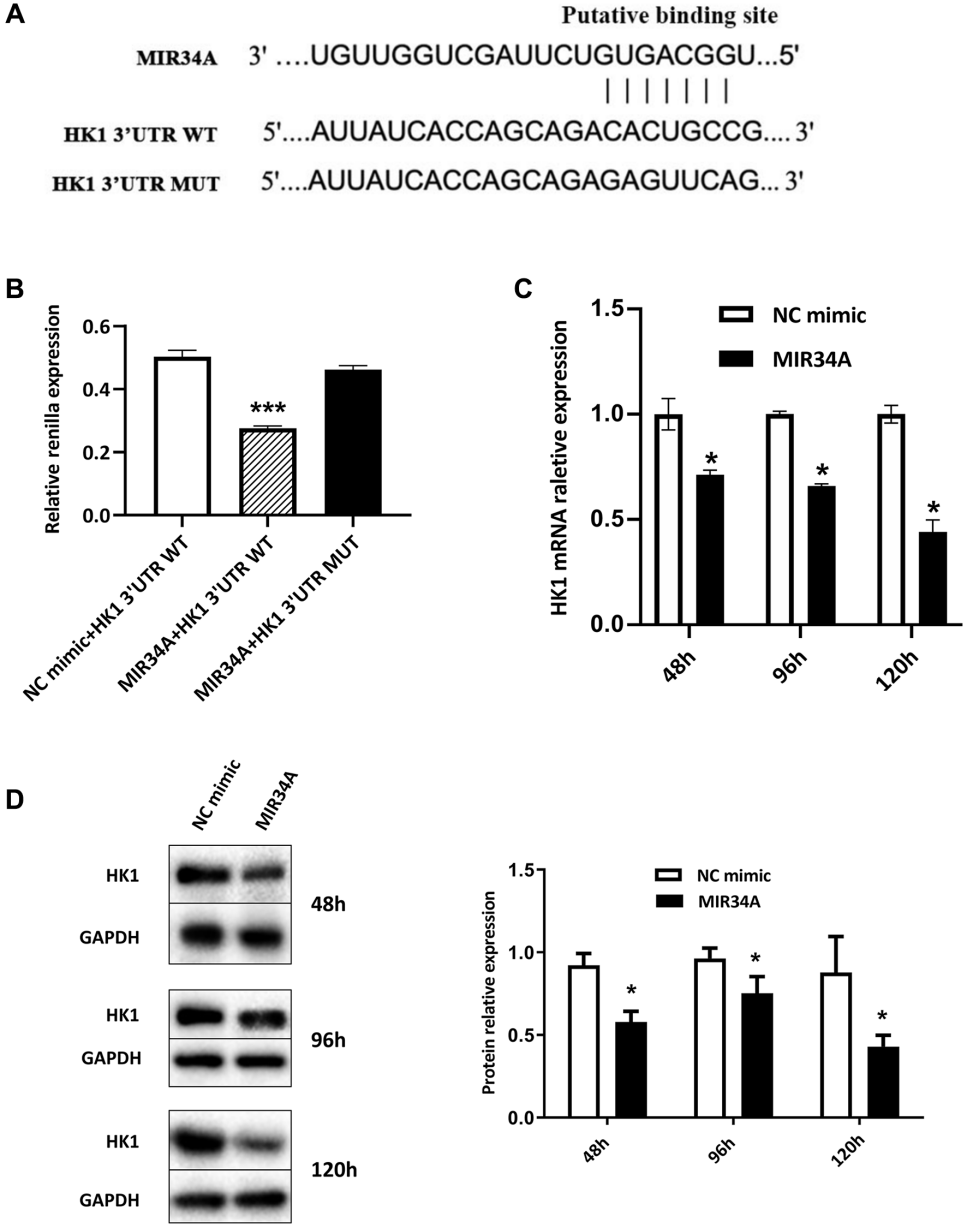
**MIR34A directly targets and represses the expression of HK1.** (**A**) A Bioinformatics-based target analysis showed that HK1 is a potential target of MIR34A. (**B**) The 293T cells were transfected with MIR34A and HK1 3’UTR WT co-transfected into 293T cells. Luciferase reporter assay showed that the luciferase activity of HK1 3′UTR-WT significantly decreased with MIR34A mimic transfection, compared to that of the NC mimic or HK1 3′UTR-mutant group. (**C**) RT-qPCR analysis was performed to detect the expression of HK1 after MIR34A mimics were transfected into SRA01/04 cells at 48 h, 96 h, and 120 h. (**D**) SRA01/04 cells were performed as C, and the expression of HK1 was detected by western blot. ^*^P < 0.05; ^***^P < 0.001.

MiRWalk was used to investigate the potential mRNAs targeted by MIR34A and find potential binding genes. The results indicated that MIR34A may bind to hexokinase 1 (HK1). TargetScan was then used to analyze the HK1 for MIR34A binding sites. We found positions 2730-2737 bp matched a single recognition sequence (Figure 2A) identified in the 3′untranslated region (3′UTR) of HK1. To verify that HK1 is directly bound by MIR34A, we constructed a luciferase reporter gene of HK1 consisting of a seed target for MIR34A in the 3′UTR and the *Renilla* luciferase gene in the plasmid psiCHECK. When MIR34A and HK1 3′UTR WT were co-transfected into 293T cells, *Renilla* luciferase gene expression significantly decreased (Figure 2B). However, this suppression effect disappeared when the seed sites were mutated. These results suggest that MIR34A can directly bind to HK1, inhibiting its expression.

To further verify our luciferase reporter results and investigate whether MIR34A could regulate the expression of HK1, we characterized the expression of HK1 when the expression of MIR34A was upregulated in SRA01/04 cells. Our data suggest that overexpression of MIR34A, significantly suppressed the expression of HK1 at 48, 96, and 120 h after treatment with MIR34A mimics based on the levels of both mRNA (Figure 2C) and protein (Figure 2D). These results indicate that HK1 is involved in the pathological development of cataracts and that this is regulated by the direct binding of MIR34A.

### Downregulation in HK1 expression suppresses the proliferation and induces the apoptosis of SRA01/04 cells

**Figure 3:**
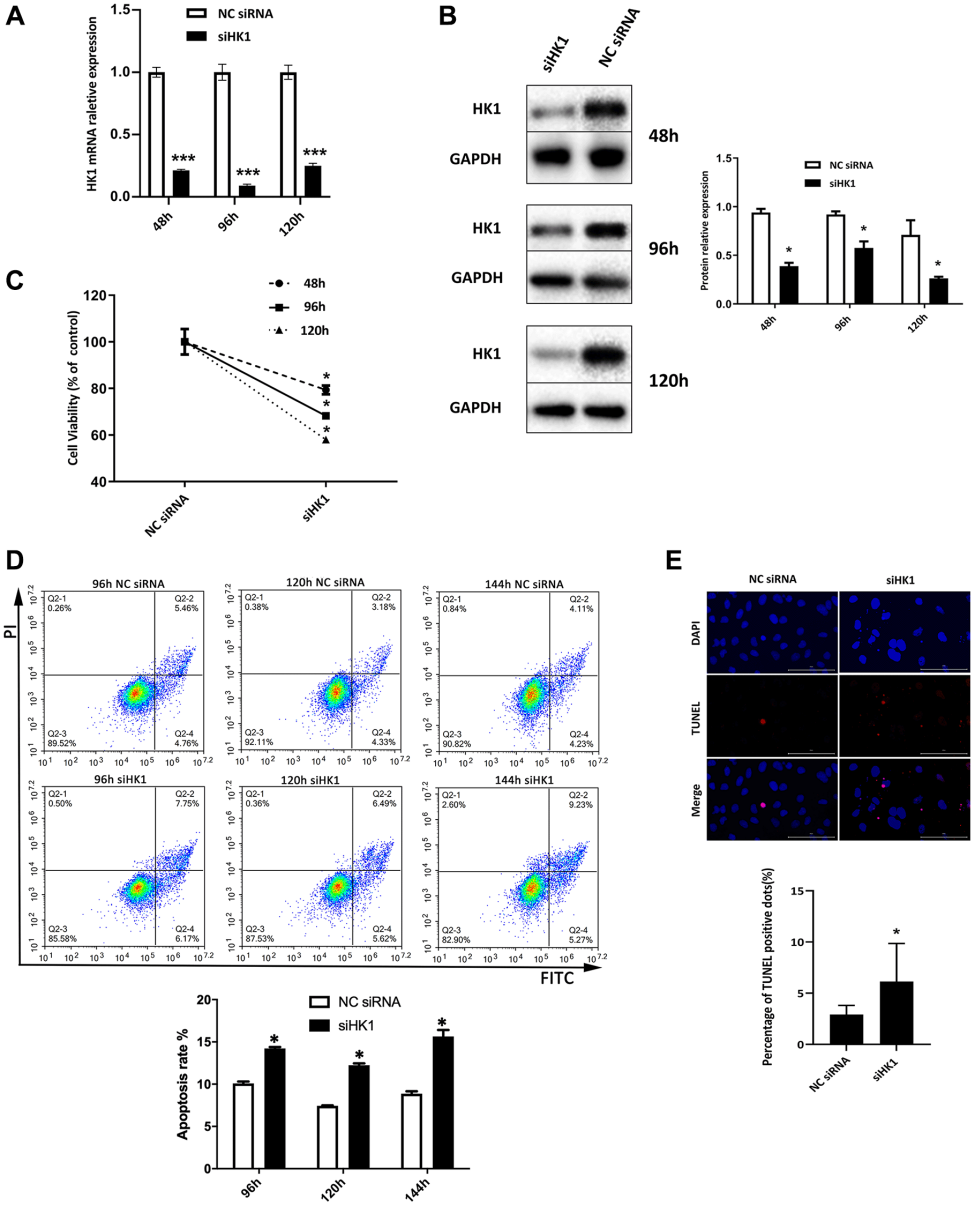
**Downregulation in HK1 expression suppresses the proliferation and induces the apoptosis of SRA01/04 cells.** (**A**) SRA01/04 cells were transfected with siRNA HK1, and the effect of siHK1 on HK1 expression in mRNA level was detected by RT-qPCR assay. (**B**) SRA01/04 cells were performed as A, and the effect of siHK1 on HK1 expression at the protein level was analyzed by western blot. (**C**) SRA01/04 cells were performed as A, the viability of cells was assessed at 48 h, 96 h, and 120 h by CCK-8 after transfected. (**D**) SRA01/04 cells were performed as A, cell apoptosis was assessed at 96 h, 120 h, and 144 h and was detected by flow cytometry. (**E**) SRA01/04 cells were performed as A, cell apoptosis at 120 h by TUNEL assay. Scale bars: 100 μm. ^*^P < 0.05; ^***^P < 0.001.

To determine whether HK1 affects the proliferation and apoptosis of SRA01/04 cells, HKI was silenced using siRNA HK1. Following transfection of siRNA HK1, the expression of HK1 of SRA01/04 cells was significantly decreased as determined at both the mRNA (Figure 3A) and protein (Figure 3B) levels. The CCK-8 assay was used to quantify SRA01/04 cell proliferation, and the results show significantly reduced cell proliferation at 48, 96, and 120 h after siRNA HK1 transfection (Figure 3C). Flow cytometry was used to measure the apoptotic rate, and the results show the apoptotic rate was significantly increased in SRA01/04 cells transfected with siRNA HK1 compared with the NC siRNA group (Figure 3D). In addition, an increase in TUNEL-positive cells was observed after the downregulation of HK1 (Figure 3E). All these results indicate that downregulating the expression of HK1 suppresses the proliferation of SRA01/04 cells and induces apoptosis.

### Mir34a modulates the opacification of the lens via the regulation of Hk1 expression

**Figure 4:**
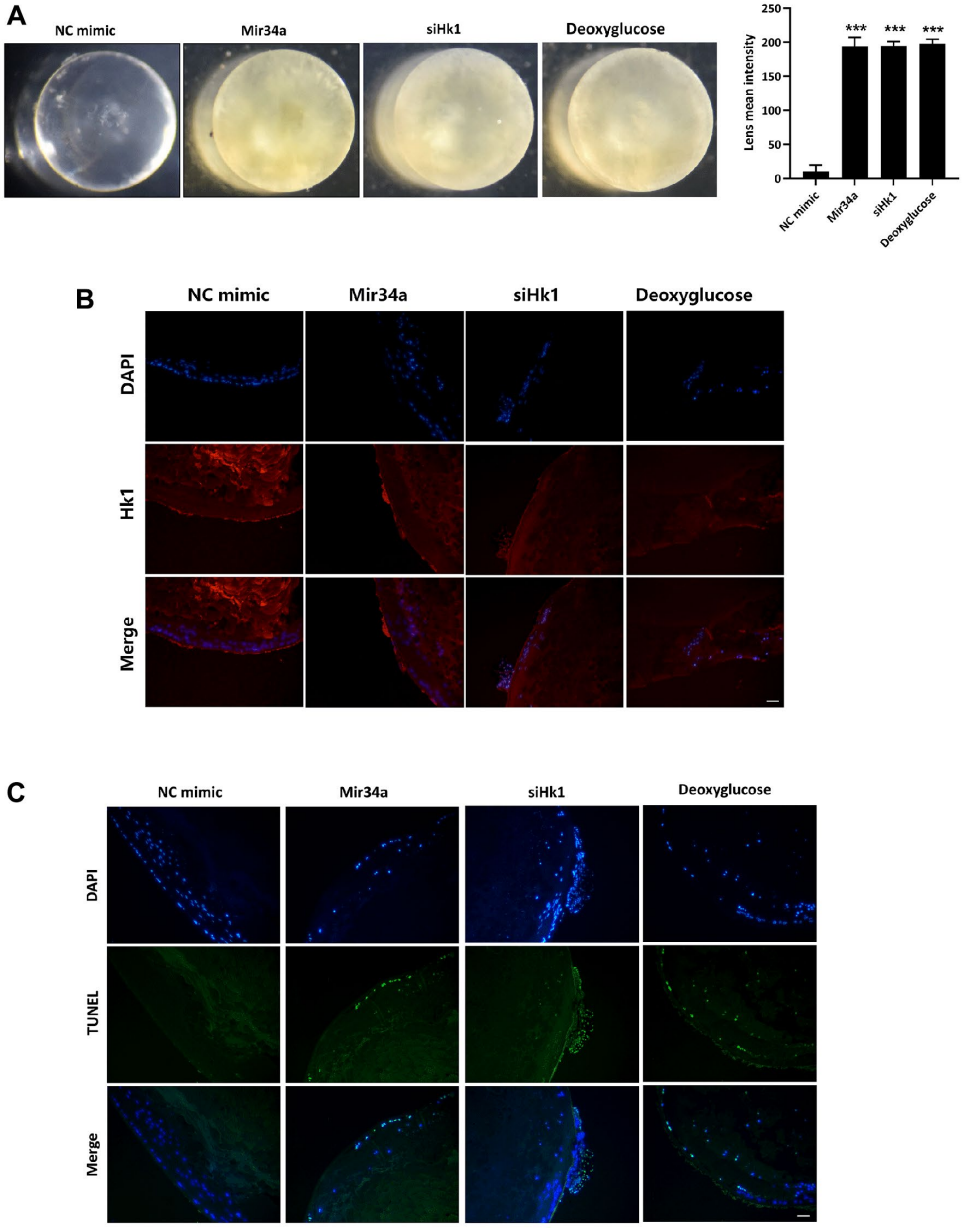
**Mir34a modulates the opacification of the lens via the regulation of Hk1 expression.** (**A**) The lens explant of the mouse was co-cultured with Mir34a, siHk1, or deoxyglucose, then the opacification of the lens was observed. (**B**) Lens explants of the mouse were performed as A, and Hk1 expression on lens epithelium was detected by immunofluorescence. Scale bars: 20 μm. (**C**) Lens explants of the mouse were performed as A, and the apoptosis of lens epithelium was detected by TUNEL. Scale bars: 20 μm. ^***^P < 0.001.

To evaluate whether Mir34a plays a crucial role in the pathological development of cataracts via regulating the expression of HK1, we cultured the mouse lens *in vitro* with or without Mir34a mimics, siHk1, or Hk1 inhibitor (deoxyglucose). As illustrated in Figure 4A, the lens explant showed more severe opacification after co-culturing with Mir34a, siHk1, and deoxyglucose than the control group (NC group) separately. Additionally, Hk1 expression on the lens epithelium was detected by immunofluorescence. The results show that Hk1 expression on the lens epithelium decreased after the lens explant was co-cultured with Mir34a mimics and siHk1, and no noticeable Hk1 changes were observed after the lens explant was co-cultured with deoxyglucose (Figure 4B). TUNEL assay was then used to characterize apoptosis in cells of the lens epithelium, which showed that TUNEL-positive cells of the lens epithelium increased after co-culture with either Mir34a mimics, siHk1, or deoxyglucose compared to the control group (Figure 4C). These results indicate that Hk1 modulates the apoptotic rate of cells in the lens epithelium and lens opacification via targeting of Hk1.

### MIR34A modulates LECs apoptosis and cataract development through the HK1/caspase 3 signaling pathway

**Figure 5:**
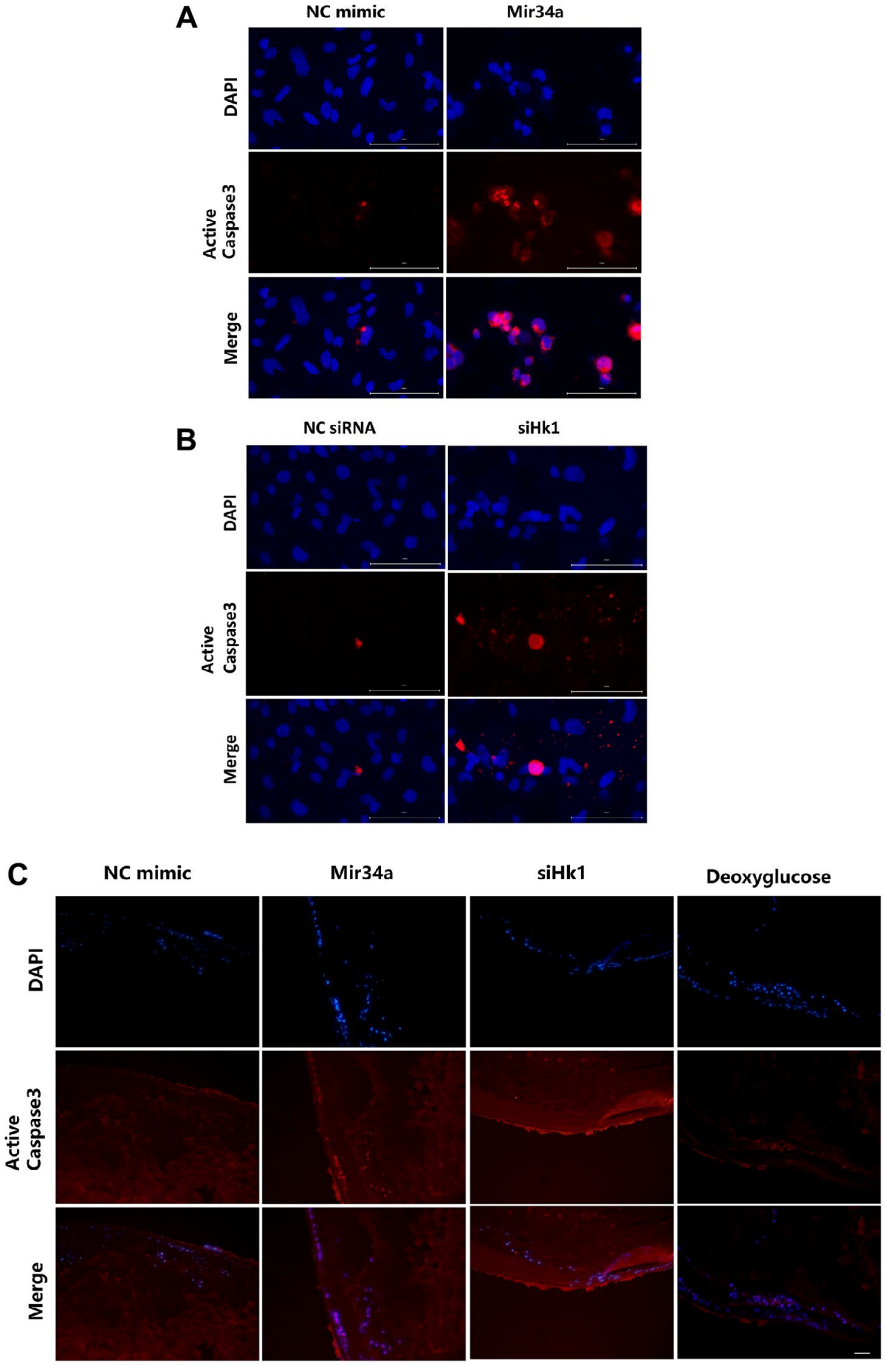
**MIR34A modulates LECs apoptosis and cataract development through the HK1/caspase 3 signaling pathway.** (**A**) SRA01/04 cells were transfected with MIR34A mimic and the expression of active caspase 3 at 120 h was detected by immunofluorescence. Scale bars: 100 μm. (**B**) SRA01/04 cells were transfected with siHK1 and the expression of active caspase 3 at 120 h was detected by immunofluorescence. Scale bars: 100 μm. (**C**) Lens explants of mice were co-cultured with Mir34a, siHk1, or deoxyglucose, and the expression of active caspase 3 of lens epithelium was detected by immunofluorescence. Scale bars: 20 μm.

Previous research has shown that caspase 3 is involved in HK1-associated cell apoptosis [27, 28]. Therefore, to determine whether MIR34A modulates LEC apoptosis and cataracts through the HK1/caspase 3 signaling pathway, we detected the expression of caspase 3 with or without MIR34A mimics or siHK1. The results show an increase in caspase 3 after treatment with MIR34A mimics (Figure 5A) and transfection of siHK1 (Figure 5B) into SRA 01/04. Additionally, we detected caspase 3 in the lens epithelium of the lens explant. We found caspase 3 to be highly expressed in the lens epithelium of the lens explant after co-culturing with either Mir34a mimics, siHk1, or deoxyglucose compared to the control group (Figure 5C). All these findings indicate that MIR34A modulates LEC apoptosis and cataract development through the HK1/caspase 3 signaling pathway.

## DISCUSSION

The exact pathogenesis of cataracts, namely in the opacification of the lens, remains unclear. It has been verified that the miR-34 family plays significant roles in many aspects, including in proliferation, apoptosis/cell survival, and senescence/aging by regulating the expression of target genes; however, its biological effects on LEC apoptosis and cataracts remain unclear. HK1, as the rate-limiting enzyme in glucose metabolism, which plays a crucial role in ATP production in the lens, was identified as one of the putative mRNA targets of MIR34A. However, its biological effects on the apoptosis of LECs and cataracts remain unclear. Specifically, our results from cultured cells and mouse lenses reveal that MIR34A modulates the HK1/caspase 3 signaling pathway and has a crucial role in LEC apoptosis and cataracts.

Increasing miRNAs have been identified to be aberrantly expressed in the process of disease development, and miRNAs are potential therapeutic targets for diseases, including cataracts [29, 30]. Previous studies have shown that MIR34A is involved in various diseases, including myocardial infarction [31], senescence [32], tuberous sclerosis complex [33], and hepatocellular carcinoma [34]. Additionally, MIR34A is abnormally expressed in some age-related diseases, such as age-associated heart failure [35], atherosclerosis [36], and posterior capsule opacification [37]. Chien et al. [38] demonstrated that MIR34A plays a crucial role in lens senescence. Consistent with a previous study, our results showed overexpression of MIR34A in the LECs of cataracts [20]. Additionally, we found that overexpression of Mir34a accelerated the opacification of the mouse lens. Han et al. [37] found MIR34A can inhibit the epithelial–mesenchymal transition of LECs via regulating Notch1 to inhibit the occurrence of posterior capsule opacification. Feng et al. [39] demonstrated MIR34A might be an inhibitor in posterior capsule opacification via regulating human LEC proliferation and migration by targeting c-Met. Additionally, Li et al. [40] found MIR34A promotes the apoptosis of human lens epithelial cells by downregulating Bcl-2 and SIRT1.

As an essential enzyme responsible for the first step of glycolysis, HKs are involved in many diseases and are controlled by miRNAs. Most previous research points to a relationship between HK2 and miRNAs, but little is known about the interaction between HK1 and miRNAs. Chen et al. [41] found that long noncoding RNA PVT1 increased the expression of HK2 by its competitive endogenous RNA activity against miR-143 in gallbladder cancer. Ren et al. found [42] circDENND4C silence suppresses glycolysis by downregulating the expression of HK2 via targeting miR-200 under hypoxia. Lin et al. [43] demonstrated that taurine regulates cell growth, metastasis, and glycolysis through the miR-455-3p/HK2 pathway in hepatocellular carcinoma patients. Recently, Zhou et al. [44] demonstrated that MIR34A could inhibit the development of hepatocellular carcinoma by regulating HK1 expression. Consistent with the previous study, our results show MIR34A inhibits HK1 expression by binding to the 3′UTR of HK1. Additionally, we found that overexpression of MIR34A inhibited the proliferation and induced the apoptosis of SRA01/04 cells via HK1 targeting. Moreover, we found that overexpressing Mir34a accelerated the opacification of the mouse lens via the regulation of Hk1 expression.

Apoptosis plays a vital role in cataract formation, both *in vitro* and *in vivo*. Research has shown that treatment of lenses with stress factors induces LEC apoptosis and leads to eventual noncongenital cataracts [45, 46]. HKs and apoptosis are closely related. Chu et al. [47] showed HK2 could increase the levels of Bcl-2 and decrease the levels of apoptosis in retinal oxidative stress injury. Additionally, Behar et al. [48] found that selectively detaching HK2 by Comp-1 (a small allosteric molecule) can reduce glycolysis and trigger apoptosis in cancer cells without affecting the expression of HK1 in normal cells. However, the observations of Sen et al. [28] suggest that dissociating HK1 from the mitochondria can induce apoptosis in human immunodeficiency virus-1 infected peripheral blood mononuclear cells. Consistent with the previous study, our results showed downregulation in the expression of HK1 significantly induced apoptosis in LECs. Moreover, we found that downregulated Hk1 accelerated the opacification of the lenses of mice.

The family of caspases plays a crucial role in apoptosis; among all the family members, activated caspase 3 is the most abundant executioner caspase [49]. Many studies have identified that the activity of caspase 3 is regulated by HKs [28, 50, 51]. In retinal epithelial cells of retinal oxidative stress injury, HK2 knockdown can induce apoptosis via an increase in cleaved caspase 3 [47]. In small pulmonary arteries, reduced expression of HK2 enhanced the expression of caspase 3 [51]. In human immunodeficiency, virus-1 infected macrophages, dissociation of HK1 from mitochondria in viral protein R transduced U937 also activated caspase 3/7 activity [28]. According to the previous study, a reduction of 50% in HK1 is severe enough to decrease the inner mitochondrial membrane (IMM) potential, and disrupt the IMM potential and mitochondrial integrity. Furtherly, a reduction of 50% in HK1 accelerated TNF-induced apoptosis by Bcl-2/Bax signal pathway [52, 53]. Additionally, when HK2 drops to 50% in the mitochondria of cardiomyocytes, caspase 9 and caspase 3 are activated, then leading to apoptosis [54]. In our research, we found that after reducing HK1 expression to less than 50% by MIR34A or siHK1, the activity of caspase 3 was increased in SRA01/04 cells and the LECs of mouse lenses. The reason why HK1 decreased by 50% was enough to cause cataracts and apoptosis might be that IMM potential and mitochondrial integrity were destroyed in LECs. Additionally, the overexpression of Mir34a and Hk1 downregulation could inhibit proliferation and induce apoptosis in LECs. Overall, these results reveal that the MIR34A–HK1/caspase 3 signaling pathway plays a vital role in cataract formation.

However, there are several limitations to our study. First, we only performed *in vitro* experiments in LECs. *In vivo*, experiments should be performed in mice to verify the role of MIR-34A and HK1 in cataracts. Many questions remain to be answered in mice, including whether the expression of HK1 is abnormal in the LECs of mice and whether regulating the expression of MIR34A and HK1 can prevent cataracts. Second, although two isomers (HK1 and HK2) are expressed in the human lens, we only detected the function of HK1 in LECs. Thus, whether HK2 is involved in cataracts requires further exploration. Third, in our whole study, we did not directly detect the enzyme activity of HK1 but only detected its expression at the mRNA level and protein level. To verify that decreased HK1 activity leads to LECs apoptosis, HK1 activity should be detected. Fourthly, to demonstrate that the caspase 3 signaling pathway plays a vital role in cataract formation, we should disrupt the expression of active caspase 3 and then observe the opacification of the lens. These questions will be further studied in our future studies.

In summary, despite the abovementioned limitations, our results demonstrate, for the first time, the HK1 mRNA is a direct target of MIR34A, whereby the high expression of MIR34A in the cataracts lens suppresses the expression of HK1. *In vitro*, the upregulation of MIR34A together with the downregulation of HK1 inhibits the proliferation, induces the apoptosis of SRA01/04 cells, and accelerates the opacification of mouse lenses via the HK1/caspase 3 signaling pathway. These findings provide a new perspective for research into the mechanisms underlying cataracts and provide a new direction for future studies of the molecular mechanisms of pathogenesis of cataracts.

## MATERIALS AND METHODS

### Study approval and material preparation

The animals were maintained and treated in accordance with the ARVO Statement for the Use of Animals in Ophthalmic and Vision Research, and the present study was approved by the animal ethics committee of Zhongshan Ophthalmic Center, Sun Yat-sen University (Guangzhou, China; approval no. ID:2020136). The mice were housed in cages (one rat/cage) maintained in standard conditions (room temperature 23 ± 2°C; relative humidity 60 ± 10%; 12 h light/dark cycle) and were fed a standard laboratory diet with ad libitum access to water.

Eight-week-old mice were intraperitoneally injected with the pentobarbital and euthanized by cervical vertebra dislocation. After enucleation, lenses were extracted from the globes using a posterior approach, washed with PBS, and then placed in a 12-well plate. Each well contained 3 mL of prewarmed bicarbonate-based medium 199 (Gibco) supplemented with penicillin (100 U/mL) and streptomycin (100 U/mL). Lens explants were cultured for 24 h and damaged and contaminated lenses were excluded. These were treated with 50 nM MiR34a agomir (Guangzhou RiboBio Co., Ltd., China), 50 nM siHk1 (Guangzhou RiboBio Co., Ltd., China), or 20 mM deoxyglucose (Beyotime Biotechnology, China). Photography, TUNEL, and immunofluorescence for caspase 3 and Hk1 were performed after the lens explant was treated for 96 h.

Lens opacity was evaluated by the severity of whole lens opacity after the MiR34a agomir, siHk1, or deoxyglucose treatment for 96 h. For the severity of whole lens opacity, lens images were captured with an Olympus D5100 camera (Nikon, Tokyo, Japan). The lens images were converted into 8-bid grayscale images and analyzed using the open-source software ImageJ [55]. Distributions of pixel intensities were plotted into a histogram, where an increase in the intensity value corresponded to the increased opacity of the lens.

### Cell culture and transfection

Both the human lens epithelial cell line SRA01/04 (kind gifts from Xuhua Tan) and 293T cells (American Type Culture Collection, Manassas, VA, USA) were incubated at 37°C in a humidified incubator containing 5% CO_2_. The 293T cells and SRA01/04 cells were, respectively, cultured in low- and high-glucose Dulbecco’s modified Eagle’s medium (DMEM; Gibco^®^ Invitrogen; Thermo Fisher Scientific, Inc.), which contained 10% FBS, 100 U/mL penicillin, and 100 U/mL streptomycin.

The potential binding sites for MIR34A within the 3′-untranslated region (UTR) of human HK1 were cloned into the dual-luciferase vector psiCHECK2 (Promega Corporation, Madison, WI, USA), and the construct was named psiCHECK-HK1. A mutant 3′-UTR fragment of HK1 with mutations in MIR34A seed binding sites was generated, which was named psiCHECK-HK1 mut. MIR34A mimics, small interfering (si)HK1, and negative control were synthesized by Guangzhou Ruibo Biological Co., Ltd. (Guangzhou, China). Plasmid and microRNA mimics were co-transfected using Lipofectamine 3000 (Invitrogen, Carlsbad, CA, USA) and RNAiMAX (Invitrogen, Carlsbad, CA, USA), respectively.

### RT-qPCR

Total RNA was extracted using TRIzol reagent (CW0580S, CoWin Biosciences, China) according to the manufacturer’s instructions. The HiScript II Q RT SuperMix for qPCR (R223-01, Vazyme, China), miRNA cDNA synthesis kit (CW2141S, Cowin Biosciences, China), and miRNA qPCR assay kit (CW2142S, Cowin Biosciences, China) were used for qPCR. The primer sequences and their targets are detailed in Table 1. The 2^−ΔΔCT^ method was used to calculate the relative expression levels of genes.

**Table 1:** List of primer sequences used for qPCR.

**Gene**	**Sequences (5′–3′)**
HK1	Forward: TATTCCCGGCGTTTCCACAA
	Reverse: GAAGTCACCATTCTCGGTCCC
GAPDH	Forward: TGACTTCAACAGCGACACCCA
	Reverse: CACCCTGTTGCTGTAGCCAAA
U6	Forward: GCTTCGGCAGCACATATACTAAAAT
	Reverse: CGCTTCACGAATTTGCGTGTCAT
Hsa-MIR34A-5p	Forward: TGGCAGTGTCTTAGCTGGTTGT
	Reverse: ACGGATGTCAACGTCACACT

### Cell viability assay

The SRA01/04 cells were seeded into 96-well cell culture plates and incubated overnight to allow attachment. After the indicated treatments, cell viability was detected using the Cell Counting Kit-8 assay (KGA317, KeyGEN BioTECH, China) according to the manufacturer’s instructions.

### Flow cytometry and TUNEL assay

Flow cytometry was used to characterize the apoptosis of SRA01/04 cells cultured in the presence of MIR34A mimic, siHK1, and their negative control at different time points of 96, 120, and 144 h. Apoptosis was tested with annexin V–fluorescein isothiocyanate/propidium iodide staining using an apoptosis detection kit (AP101-100-kit, MULTI SCIENCES, China). The detailed procedures were performed according to the manufacturer’s instructions, and the samples were analyzed by flow cytometry (NovoCyte 2060R, ACEA Biosciences, Inc., USA).

Cells were seeded into 96-well cell culture plates. After transfection for 120 h, the cells were fixed with 4% paraformaldehyde and permeabilized with 0.1% Triton X-100. A terminal deoxynucleotidyl transferase dUTP nick end labeling (TUNEL) assay was then conducted based on the manufacturer’s instructions (Beyotime Biotechnology, China).

### Dual-luciferase reporter assay

Cells from line 293T were plated in 24-well plates for co-transfection of either psiCHECK-HK1 or psiCHECK-HK1 mut in the presence of MIR34A or the mimic control. At 48 h after transfection, a Tecan Safire Microplate Reader II (Tecan Group, Ltd., Mannedorf), Dual-Glo luciferase assay system (Promega Corporation, Switzerland) was used to detect fluorescence and *Renilla* luciferase activity.

### Western blot

Cell lysates were harvested in a complete lysis buffer (Beyotime, Shanghai, China). The cell sample proteins were separated by SDS-PAGE (Beyotime Biotechnology, P0012A) and electroblotted to a polyvinylidene difluoride membrane (PVDF; Millipore, IPVH00010) as previously described [56]. For all cases, the protein blot was blocked for one hour at room temperature with a blocking solution (5% nonfat dried milk) and tris-buffered saline and Tween 20. The membranes were incubated at 4°C overnight with dilutions of antibodies against HK1 (19662-1-ap, 1:500, Proteintech, USA) or GAPDH (TA-08, 1:2000, ZSGB-Bio, China). Following incubation with the corresponding secondary antibodies, enhanced chemiluminescence (RJ239676, Thermo Fisher Scientific, Inc., USA) was used for visualization.

### Immunofluorescence analysis

Immunofluorescence analysis was performed on the SRA01/04 cells as previously described [57]. Cell samples were fixed and then incubated at 4°C overnight with anti-active caspase 3 (Asp175) antibody (#9661; 1:400; Cell Signaling Technology, Inc., Danvers, MA, USA) or HK1 (#2024; 1:200; Cell Signaling Technology, Inc., Danvers, MA, USA). Subsequently, Alexa Fluor 647-labeled goat anti-rabbit IgG (Beyotime Biotechnology, China) was added, and the nuclei were stained with 4′,6-diamidino-2-phenylindole (Polysciences, 09224-10). The cells were observed using fluorescence microscopy.

### Statistical analysis

Experiments were conducted three times with independent biological and technical replicates. One-way analysis of variance was used to analyze and compare the differences between groups. Data are expressed as the mean ± standard deviation from at least three independent experiments. A *p*-value of less than 0.05 was considered to indicate a statistically significant difference.

## References

[1] Liu YC, Wilkins M, Kim T, Malyugin B, Mehta JS. Cataracts. Lancet. 2017; 390:600–12. 10.1016/S0140-6736(17)30544-5 28242111

[2] Patel K, Patel S. Angle-closure glaucoma. Dis Mon. 2014; 60:254–62. 10.1016/j.disamonth.2014.03.005 24906670

[3] Bievel-Rădulescu R, Tăbăcaru B, Stanca HT. Lens-induced uveitis in a patient with hypermature cataract. Rom J Ophthalmol. 2021; 65:300–6. 10.22336/rjo.2021.62 35036658 PMC8697789

[4] Wu S, Yu X, Dai Q, Fu Y, Lin X. Corneal decompensation due to spontaneous absorption of lens and anterior dislocation of lens capsule: A case report. Medicine (Baltimore). 2019; 98:e18417. 10.1097/MD.0000000000018417 31852166 PMC6922511

[5] Louison S, Blanc J, Pallot C, Alassane S, Praudel A, Bron AM, Creuzot-Garcher C. Visual outcomes and complications of congenital cataract surgery. J Fr Ophtalmol. 2019; 42:368–74. 10.1016/j.jfo.2018.10.007 30898370

[6] Zeng K, Feng QG, Lin BT, Ma DH, Liu CM. Effects of microRNA-211 on proliferation and apoptosis of lens epithelial cells by targeting *SIRT1* gene in diabetic cataract mice. Biosci Rep. 2017; 37:BSR20170695. 10.1042/BSR20170695 28679650 PMC5529207

[7] Martinez G, de Iongh RU. The lens epithelium in ocular health and disease. Int J Biochem Cell Biol. 2010; 42:1945–63. 10.1016/j.biocel.2010.09.012 20883819

[8] Nibourg LM, Gelens E, Kuijer R, Hooymans JM, van Kooten TG, Koopmans SA. Prevention of posterior capsular opacification. Exp Eye Res. 2015; 136:100–15. 10.1016/j.exer.2015.03.011 25783492

[9] Meduri A, Bergandi L, Oliverio GW, Rechichi M, Acri G, Perroni P, Silvagno F, Aragona P. The cold eye irrigation BSS solution used during phacoemulsification reduces post-surgery patients discomfort preventing the inflammation. Eur J Ophthalmol. 2021; 32:911–7. 10.1177/11206721211018377 34011203

[10] Bouffard MA, Cestari DM. Diplopia after Cataract Extraction. Semin Ophthalmol. 2018; 33:11–6. 10.1080/08820538.2017.1353806 28990829

[11] Barnes PJ, Baker J, Donnelly LE. Cellular Senescence as a Mechanism and Target in Chronic Lung Diseases. Am J Respir Crit Care Med. 2019; 200:556–64. 10.1164/rccm.201810-1975TR 30860857

[12] Farooqi AA, Tabassum S, Ahmad A. MicroRNA-34a: A Versatile Regulator of Myriads of Targets in Different Cancers. Int J Mol Sci. 2017; 18:2089. 10.3390/ijms18102089 29036883 PMC5666771

[13] John A, Kubosumi A, Reddy PH. Mitochondrial MicroRNAs in Aging and Neurodegenerative Diseases. Cells. 2020; 9:1345. 10.3390/cells9061345 32481587 PMC7349858

[14] Jun S, Datta S, Wang L, Pegany R, Cano M, Handa JT. The impact of lipids, lipid oxidation, and inflammation on AMD, and the potential role of miRNAs on lipid metabolism in the RPE. Exp Eye Res. 2019; 181:346–55. 10.1016/j.exer.2018.09.023 30292489 PMC6443454

[15] Niemiec SM, Louiselle AE, Liechty KW, Zgheib C. Role of microRNAs in Pressure Ulcer Immune Response, Pathogenesis, and Treatment. Int J Mol Sci. 2020; 22:64. 10.3390/ijms22010064 33374656 PMC7793489

[16] Raucci A, Macrì F, Castiglione S, Badi I, Vinci MC, Zuccolo E. MicroRNA-34a: the bad guy in age-related vascular diseases. Cell Mol Life Sci. 2021; 78:7355–78. 10.1007/s00018-021-03979-4 34698884 PMC8629897

[17] Raucci A, Vinci MC. miR-34a: A Promising Target for Inflammaging and Age-Related Diseases. Int J Mol Sci. 2020; 21:8293. 10.3390/ijms21218293 33167452 PMC7663903

[18] Chen P, Chen F, Lei J, Li Q, Zhou B. Activation of the miR-34a-Mediated SIRT1/mTOR Signaling Pathway by Urolithin A Attenuates D-Galactose-Induced Brain Aging in Mice. Neurotherapeutics. 2019; 16:1269–82. 10.1007/s13311-019-00753-0 31420820 PMC6985387

[19] Huang Q, Zheng Y, Ou Y, Xiong H, Yang H, Zhang Z, Chen S, Ye Y. miR-34a/Bcl-2 signaling pathway contributes to age-related hearing loss by modulating hair cell apoptosis. Neurosci Lett. 2017; 661:51–6. 10.1016/j.neulet.2017.07.044. Retraction in: Neurosci Lett. 2019; 707:134290. 10.1016/j.neulet.2017.07.044 28756190

[20] Xiang W, Lin H, Wang Q, Chen W, Liu Z, Chen H, Zhang H, Chen W. miR-34a suppresses proliferation and induces apoptosis of human lens epithelial cells by targeting E2F3. Mol Med Rep. 2016; 14:5049–56. 10.3892/mmr.2016.5901 27840975 PMC5355663

[21] Ho PC, Bihuniak JD, Macintyre AN, Staron M, Liu X, Amezquita R, Tsui YC, Cui G, Micevic G, Perales JC, Kleinstein SH, Abel ED, Insogna KL, et al. Phosphoenolpyruvate Is a Metabolic Checkpoint of Anti-tumor T Cell Responses. Cell. 2015; 162:1217–28. 10.1016/j.cell.2015.08.012 26321681 PMC4567953

[22] Wiel C, Le Gal K, Ibrahim MX, Jahangir CA, Kashif M, Yao H, Ziegler DV, Xu X, Ghosh T, Mondal T, Kanduri C, Lindahl P, Sayin VI, Bergo MO. BACH1 Stabilization by Antioxidants Stimulates Lung Cancer Metastasis. Cell. 2019; 178:330–45.e22. 10.1016/j.cell.2019.06.005 31257027

[23] Chylack LT Jr. Human lens hexokinase. Exp Eye Res. 1973; 15:225–33. 10.1016/0014-4835(73)90123-1 4632512

[24] McNulty R, Wang H, Mathias RT, Ortwerth BJ, Truscott RJ, Bassnett S. Regulation of tissue oxygen levels in the mammalian lens. J Physiol. 2004; 559:883–98. 10.1113/jphysiol.2004.068619 15272034 PMC1665185

[25] Helbig H, Hinz JP, Kellner U, Foerster MH. Oxygen in the anterior chamber of the human eye. Ger J Ophthalmol. 1993; 2:161–4. 8334391

[26] Hockwin O. Age changes of lens metabolism. Altern Entwickl Aging Dev. 1971; 1:95–129. 4953133

[27] Wang ZC, Wang ZZ, Ma HJ, Wang CC, Wang HT. Attenuation of the hypoxia-induced miR-34a protects cardiomyocytes through maintenance of glucose metabolism. Biochem Biophys Res Commun. 2018; 498:375–81. 10.1016/j.bbrc.2017.06.030 28709867

[28] Sen S, Kaminiski R, Deshmane S, Langford D, Khalili K, Amini S, Datta PK. Role of hexokinase-1 in the survival of HIV-1-infected macrophages. Cell Cycle. 2015; 14:980–9. 10.1080/15384101.2015.1006971 25602755 PMC4612415

[29] Varma SD, Kovtun S, Hegde K, Yin J, Ramnath J. Effect of high sugar levels on miRNA expression. Studies with galactosemic mice lenses. Mol Vis. 2012; 18:1609–18. 22736950 PMC3380916

[30] Wang S, Guo C, Yu M, Ning X, Yan B, Zhao J, Yang A, Yan H. Identification of H_2_O_2_ induced oxidative stress associated microRNAs in HLE-B3 cells and their clinical relevance to the progression of age-related nuclear cataract. BMC Ophthalmol. 2018; 18:93. 10.1186/s12886-018-0766-6 29653565 PMC5899325

[31] Zhang F, Wang K, Gao F, Xuan Y, Liu X, Zhang Z. Resveratrol Pretreatment Improved Heart Recovery Ability of Hyperglycemic Bone Marrow Stem Cells Transplantation in Diabetic Myocardial Infarction by Down-Regulating MicroRNA-34a. Front Pharmacol. 2021; 12:632375. 10.3389/fphar.2021.632375 34177568 PMC8223511

[32] Pi C, Ma C, Wang H, Sun H, Yu X, Gao X, Yang Y, Sun Y, Zhang H, Shi Y, Li Y, Li Y, He X. MiR-34a suppression targets Nampt to ameliorate bone marrow mesenchymal stem cell senescence by regulating NAD^+^-Sirt1 pathway. Stem Cell Res Ther. 2021; 12:271. 10.1186/s13287-021-02339-0 33957971 PMC8101138

[33] Korotkov A, Sim NS, Luinenburg MJ, Anink JJ, van Scheppingen J, Zimmer TS, Bongaarts A, Broekaart DWM, Mijnsbergen C, Jansen FE, Van Hecke W, Spliet WGM, van Rijen PC, et al. MicroRNA-34a activation in tuberous sclerosis complex during early brain development may lead to impaired corticogenesis. Neuropathol Appl Neurobiol. 2021; 47:796–811. 10.1111/nan.12717 33942341 PMC8519131

[34] Sun X, Zhu H, Cao R, Zhang J, Wang X. BACH1 is transcriptionally inhibited by TET1 in hepatocellular carcinoma in a microRNA-34a-dependent manner to regulate autophagy and inflammation. Pharmacol Res. 2021; 169:105611. 10.1016/j.phrs.2021.105611 33878446

[35] Boon RA, Iekushi K, Lechner S, Seeger T, Fischer A, Heydt S, Kaluza D, Tréguer K, Carmona G, Bonauer A, Horrevoets AJ, Didier N, Girmatsion Z, et al. MicroRNA-34a regulates cardiac ageing and function. Nature. 2013; 495:107–10. 10.1038/nature11919 23426265

[36] Gatsiou A, Georgiopoulos G, Vlachogiannis NI, Pfisterer L, Fischer A, Sachse M, Laina A, Bonini F, Delialis D, Tual-Chalot S, Zormpas E, Achangwa R, Jiang L, et al. Additive contribution of microRNA-34a/b/c to human arterial ageing and atherosclerosis. Atherosclerosis. 2021; 327:49–58. 10.1016/j.atherosclerosis.2021.05.005 34038763

[37] Han R, Hao P, Wang L, Li J, Shui S, Wang Y, Ying M, Liu J, Tang X, Li X. MicroRNA-34a inhibits epithelial-mesenchymal transition of lens epithelial cells by targeting Notch1. Exp Eye Res. 2019; 185:107684. 10.1016/j.exer.2019.05.024 31158382

[38] Chien KH, Chen SJ, Liu JH, Chang HM, Woung LC, Liang CM, Chen JT, Lin TJ, Chiou SH, Peng CH. Correlation between microRNA-34a levels and lens opacity severity in age-related cataracts. Eye (Lond). 2013; 27:883–8. 10.1038/eye.2013.90 23661155 PMC3709404

[39] Feng D, Zhu N, Yu C, Lou D. MicroRNA-34a suppresses human lens epithelial cell proliferation and migration via downregulation of c-Met. Clin Chim Acta. 2019; 495:326–30. 10.1016/j.cca.2019.04.060 30980790

[40] Li QL, Zhang HY, Qin YJ, Meng QL, Yao XL, Guo HK. MicroRNA-34a promoting apoptosis of human lens epithelial cells through down-regulation of B-cell lymphoma-2 and silent information regulator. Int J Ophthalmol. 2016; 9:1555–60. 10.18240/ijo.2016.11.04 27990356 PMC5145081

[41] Chen J, Yu Y, Li H, Hu Q, Chen X, He Y, Xue C, Ren F, Ren Z, Li J, Liu L, Duan Z, Cui G, Sun R. Long non-coding RNA PVT1 promotes tumor progression by regulating the miR-143/HK2 axis in gallbladder cancer. Mol Cancer. 2019; 18:33. 10.1186/s12943-019-0947-9 30825877 PMC6397746

[42] Ren S, Liu J, Feng Y, Li Z, He L, Li L, Cao X, Wang Z, Zhang Y. Knockdown of circDENND4C inhibits glycolysis, migration and invasion by up-regulating miR-200b/c in breast cancer under hypoxia. J Exp Clin Cancer Res. 2019; 38:388. 10.1186/s13046-019-1398-2 31488193 PMC6727545

[43] Lin YH, Wu MH, Huang YH, Yeh CT, Cheng ML, Chi HC, Tsai CY, Chung IH, Chen CY, Lin KH. Taurine up-regulated gene 1 functions as a master regulator to coordinate glycolysis and metastasis in hepatocellular carcinoma. Hepatology. 2018; 67:188–203. 10.1002/hep.29462 28802060

[44] Zhou Y, Liu K, Liu Y, Tan L. Retracted: MicroRNA-34a inhibit hepatocellular carcinoma progression by repressing hexokinase-1. J Cell Biochem. 2019; 120:7147–53. 10.1002/jcb.27988. Retraction in: J Cell Biochem. 2022; 123:494. 10.1002/jcb.27988 30474301

[45] Zhang L, Yan Q, Liu JP, Zou LJ, Liu J, Sun S, Deng M, Gong L, Ji WK, Li DW. Apoptosis: its functions and control in the ocular lens. Curr Mol Med. 2010; 10:864–75. 10.2174/156652410793937741 21091420

[46] Yan Q, Liu JP, Li DW. Apoptosis in lens development and pathology. Differentiation. 2006; 74:195–211. 10.1111/j.1432-0436.2006.00068.x 16759286

[47] Chu L, Xiao L, Xu B, Xu J. Dissociation of HKII in retinal epithelial cells induces oxidative stress injury in the retina. Int J Mol Med. 2019; 44:1377–87. 10.3892/ijmm.2019.4304 31432102 PMC6713434

[48] Behar V, Pahima H, Kozminsky-Atias A, Arbel N, Loeb E, Herzberg M, Becker OM. A Hexokinase 2 Modulator for Field-Directed Treatment of Experimental Actinic Keratoses. J Invest Dermatol. 2018; 138:2635–43. 10.1016/j.jid.2018.05.028 29908149

[49] Beroske L, Van den Wyngaert T, Stroobants S, Van der Veken P, Elvas F. Molecular Imaging of Apoptosis: The Case of Caspase-3 Radiotracers. Int J Mol Sci. 2021; 22:3948. 10.3390/ijms22083948 33920463 PMC8069194

[50] Yoo ID, Park MW, Cha HW, Yoon S, Boonpraman N, Yi SS, Moon JS. Elevated CLOCK and BMAL1 Contribute to the Impairment of Aerobic Glycolysis from Astrocytes in Alzheimer's Disease. Int J Mol Sci. 2020; 21:7862. 10.3390/ijms21217862 33114015 PMC7660350

[51] Li B, He W, Ye L, Zhu Y, Tian Y, Chen L, Yang J, Miao M, Shi Y, Azevedo HS, Ma Z, Hao K. Targeted Delivery of Sildenafil for Inhibiting Pulmonary Vascular Remodeling. Hypertension. 2019; 73:703–11. 10.1161/HYPERTENSIONAHA.118.11932 30636546

[52] Schindler A, Foley E. Hexokinase 1 blocks apoptotic signals at the mitochondria. Cell Signal. 2013; 25:2685–92. 10.1016/j.cellsig.2013.08.035 24018046

[53] Schindler A, Foley E. A functional RNAi screen identifies hexokinase 1 as a modifier of type II apoptosis. Cell Signal. 2010; 22:1330–40. 10.1016/j.cellsig.2010.04.010 20460151

[54] Kim KW, Kim SW, Lim S, Yoo KJ, Hwang KC, Lee S. Neutralization of hexokinase 2-targeting miRNA attenuates the oxidative stress-induced cardiomyocyte apoptosis. Clin Hemorheol Microcirc. 2021; 78:57–68. 10.3233/CH-200924 33523042

[55] Miyashita T, Senshu M, Ibi K, Yamanaka H, Nejishima H, Fukami T, Nakajima M. Evaluation of lens opacity due to inhibition of cholesterol biosynthesis using rat lens explant cultures. Toxicology. 2022; 465:153064. 10.1016/j.tox.2021.153064 34890705

[56] Feng L, Liang L, Zhang S, Yang J, Yue Y, Zhang X. HMGB1 downregulation in retinal pigment epithelial cells protects against diabetic retinopathy through the autophagy-lysosome pathway. Autophagy. 2022; 18:320–39. 10.1080/15548627.2021.1926655 34024230 PMC8942416

[57] He L, Zhang N, Wang L, Du L, Li C, Li Y, Li X, Zhu X, Lu Q, Yin X. Quercetin Inhibits AQP1 Translocation in High-Glucose-Cultured SRA01/04 Cells Through PI3K/Akt/mTOR Pathway. Curr Mol Pharmacol. 2021; 14:587–96. 10.2174/1874467213666200908120501 32900356

